# Decoding time-resolved neural representations of orientation ensemble perception

**DOI:** 10.3389/fnins.2024.1387393

**Published:** 2024-08-01

**Authors:** Ryuto Yashiro, Masataka Sawayama, Kaoru Amano

**Affiliations:** Graduate School of Information Science and Technology, The University of Tokyo, Tokyo, Japan

**Keywords:** EEG, decoding, inverted encoding model, multivariate pattern analysis, orientation, ensemble perception

## Abstract

The visual system can compute summary statistics of several visual elements at a glance. Numerous studies have shown that an ensemble of different visual features can be perceived over 50–200 ms; however, the time point at which the visual system forms an accurate ensemble representation associated with an individual’s perception remains unclear. This is mainly because most previous studies have not fully addressed time-resolved neural representations that occur during ensemble perception, particularly lacking quantification of the representational strength of ensembles and their correlation with behavior. Here, we conducted orientation ensemble discrimination tasks and electroencephalogram (EEG) recordings to decode orientation representations over time while human observers discriminated an average of multiple orientations. We modeled EEG signals as a linear sum of hypothetical orientation channel responses and inverted this model to quantify the representational strength of orientation ensemble. Our analysis using this inverted encoding model revealed stronger representations of the average orientation over 400–700 ms. We also correlated the orientation representation estimated from EEG signals with the perceived average orientation reported in the ensemble discrimination task with adjustment methods. We found that the estimated orientation at approximately 600–700 ms significantly correlated with the individual differences in perceived average orientation. These results suggest that although ensembles can be quickly and roughly computed, the visual system may gradually compute an orientation ensemble over several hundred milliseconds to achieve a more accurate ensemble representation.

## 1 Introduction

The visual system possesses remarkable abilities to rapidly extract the information required for specific situations from a myriad of inputs from the environment, including outlier detection among similar items and gist perception of a briefly presented picture. Ensemble perception, an ability to extract summary statistics of multiple elements (Whitney and Yamanashi Leib, 2018), is one of these rapid and implicit visual phenomena, which occurs in different dimensions from low (Parkes et al., 2001; Bauer, 2009; Solomon, 2010) to high-level features (Haberman et al., 2009; Yamanashi Leib et al., 2016). This notion is supported by psychophysical experiments in which humans accurately judge the mean feature of multiple elements even with a stimulus duration of approximately 50 ms (Chong and Treisman, 2003; Robitaille and Harris, 2011; Yamanashi Leib et al., 2016), which is comparable to and sometimes beyond the temporal resolution of individual item recognition. Indeed, human observers who successfully perceive an ensemble of a stimulus set fail to report changes in the appearance of individual elements in that set (Ariely, 2001; Haberman and Whitney, 2011). Consistent with these studies, recent neuroimaging studies have successfully discriminated between two different stimulus groups by applying multivariate pattern analyses to EEG evoked signals at approximately 100 ms post-stimulus, earlier than the emergence of the neural representation of individual items in the group (Roberts et al., 2019; Epstein and Emmanouil, 2021).

However, such rapid processing does not suffice for accurate ensemble perception achieved by a human observer. Ensemble representation could be gradually refined over time due to iterative feedforward and feedback processing along the hierarchy of multiple areas in the brain (Hochstein and Ahissar, 2002). Notably, some studies have supported this hypothesis as the accuracy of ensemble discrimination increases with the increasing duration of the stimulus (Li et al., 2016; Epstein et al., 2020). The enhanced accuracy may be achieved by computing a weighted average of multiple elements, potentially discounting the effect of outliers that impair accurate ensemble computation (Haberman and Whitney, 2010; de Gardelle and Summerfield, 2011; Li et al., 2017; Lau and Brady, 2018; Epstein et al., 2020; Pascucci et al., 2021; Tiurina et al., 2024). This is plausible given that the visual system is sensitive to outliers in multiple objects immediately after stimulus onset (Cant and Xu, 2020).

Therefore, although the visual system can quickly estimate an ensemble through a feedforward sweep from low to high visual areas, it is reasonable to assume that such crude ensemble representation evolves into a more refined ensemble representation (corresponding to observers’ perception) by processing multiple visual elements through feedforward-feedback loops over a longer period. This idea raises the following questions: when is ensemble perception fully formed in the brain? How does visual representation change over time, leading to the refined ensemble representation?

Crucially, previous studies did not fully address these questions. Most studies on ensemble perception have used only behavioral measurements or neural signals with limited temporal resolution (Cant and Xu, 2012; Im et al., 2017; Tark et al., 2021); therefore, the temporal dynamics of the neural representation during ensemble perception remains unclear. On the other hand, some studies employed EEG multivariate pattern analyses and discussed the timing of ensemble perception based on a time point at which different stimulus sets were significantly classified from EEG patterns (Roberts et al., 2019; Epstein and Emmanouil, 2021). However, successful binary classification of different stimulus sets at one-time point does not necessarily mean that ensemble representation is fully formed at that time. More specifically, it is possible that non-ensemble processing, such as sampling of different individual elements within each set, may result in the difference in overall EEG patterns across these stimulus sets, thereby leading to significant binary classification. Therefore, we argue that another approach is needed to address the questions mentioned earlier: (1) estimating temporal changes in the representational strength of individual elements and ensembles from EEG signals during ensemble perception of those elements, and (2) understanding when the representation corresponding to an observer’s ensemble perception strongly emerges.

In this study, we combined EEG recording with an ensemble judgment task using a set of orientation stimuli that are decoded from EEG/MEG signals (Cichy et al., 2015; Pantazis et al., 2018; Hajonides et al., 2021). Specifically, we decoded the representational strength of individual and ensemble orientations over time by constructing encoding models within a single orientation discrimination task and inverting these models to apply to EEG signals during an orientation ensemble discrimination task (i.e., inverted encoding model) (Brouwer and Heeger, 2009, 2011; Sprague et al., 2015; Oh et al., 2019). Additionally, we identified time points when correlation became high between the orientation representation estimated from the inverted encoding model and the perceived ensemble orientation measured with adjustment methods. This analysis allowed us to precisely understand the timing of the representation corresponding to an observer’s ensemble perception. We found that the strong representation of orientation ensembles emerged over 400–700 ms after stimulus onset. Furthermore, the orientation estimated from inverted encoding models correlated with individual differences in perceived average orientation at approximately 600–700 ms. These results suggest that although ensembles can be quickly and roughly estimated, the visual system may gradually compute an orientation ensemble over several hundred milliseconds to achieve a refined ensemble representation.

## 2 Methods

### 2.1 Participants

Fifteen paid volunteers were initially recruited for three experiments. The sample size was selected to match that of previous studies with similar methods (Roberts et al., 2019). One participant declined to participate in one of the three experiments and was excluded from the study. Analysis of the data from the remaining fourteen participants showed that three participants did not achieve above-chance orientation decoding accuracy at any time point, making a cross-decoding analysis (see below for more details) infeasible for these participants. Consequently, these four participants were excluded from the study and the results for the remaining eleven participants is presented (age: mean ± SD = 25.0 ± 3.84 years) [Similar individual differences in EEG decoding accuracy have also been reported in previous studies (Simanova et al., 2010)]. All participants had normal or corrected-to-normal vision. All experiments were approved by the Ethics Committee of the University of Tokyo and conducted in accordance with the guidelines of the Declaration of Helsinki. Written informed consent was obtained from all participants.

### 2.2 Visual stimuli

**Figure 1:**
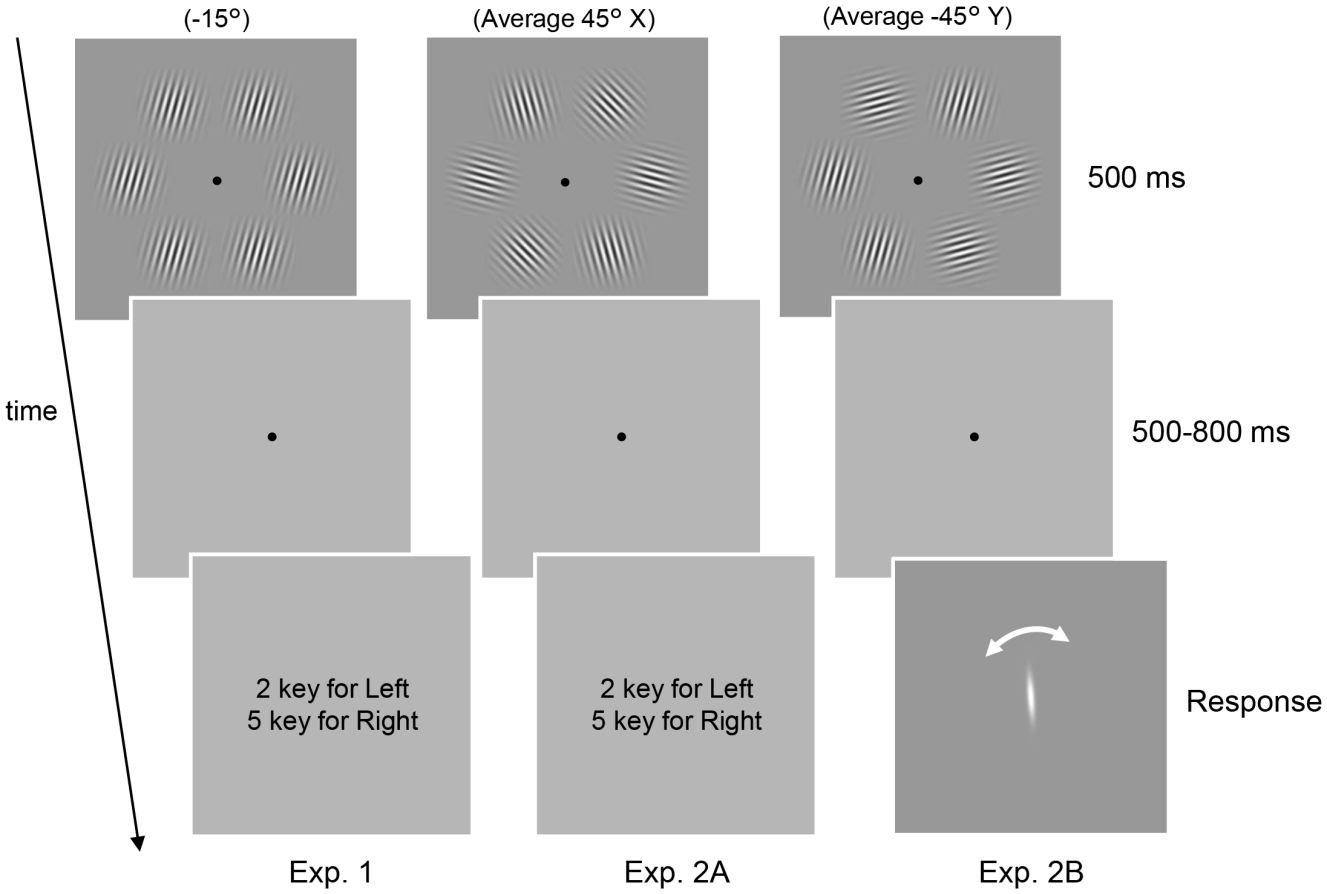
Schematic of the psychophysical experiments. In all three experiments, six Gabor patterns presented in a circle were used. The task was to report the tilt of the patterns (Experiment 1) and the average orientation (Experiment 2A and 2B). In Experiment 2B, the perceived average orientation was directly measured using adjustment methods rather than the 2AFC task.

Six Gabor patterns with a diameter of 4.8 deg. (spatial frequency: 2.0 c/deg.; Michelson contrast: 0.99) were presented against a gray background with an average luminance of 109.1 cd/m^2^ (Figure 1). The orientation of the Gabor patterns was determined according to the aim of each experiment, as explained in the following section, and each Gabor had a random phase on each trial. The center of the six Gabor patterns was located at a circle 5.8 deg away from the fixation point. The stimulus was presented on a 24-inch LCD monitor (BenQ GW2480B) at a viewing distance of 100 cm.

### 2.3 Experimental design

Two types of experiments (Experiments 1 and 2) were conducted. Experiment 1 comprised of an EEG experiment with Gabor patterns of a single uniform orientation. Participants were presented with six Gabor patterns for 500 ms (Figure 1, left panel, Experiment 1). After a blank period of 500–800 ms, the participants were asked to judge whether the patterns were tilted clockwise or counterclockwise relative to the vertical by pressing one of two buttons. The next trial was initiated 1,500 ms after the response. All six patterns had the same orientation, which was randomly determined in each trial from six orientations ranging between −75° to 75° in 30° increments, with 0° corresponding to the vertical. The response keys were randomly alternated between blocks. Participants completed 8 blocks of 120 trials, apart from one participant who only completed 6 blocks due to time constraints.

Experiment 2 consisted of EEG and behavioral experiments (Experiments 2A and 2B, respectively) with Gabor patterns with multiple orientations. In both Experiments 2A and 2B, participants were presented with a stimulus set of six Gabor patterns with different orientations (Figure 1, top center and right panels, Table 1: four stimulus sets used in the experiment). Two stimulus sets had an average orientation of 45° while the others possessed an orientation of −45°. As we were interested in how the decoding of the average orientation (45° or −45°) is affected by the presence of 45° or −45° patterns in the set, we created stimulus sets with and without elements corresponding to the average (w and w/o, respectively) for each average orientation. In each trial, participants were randomly presented with one of the sets for 500 ms. The position of each element in the set varied randomly between trials.

**Table 1:** Four stimulus sets used in Experiment 2.

Name	Components
Average 45° w	15°, 15°, 45°, 45°, 75°, 75°
Average 45° w/o	15°, 15°, 15°, 75°, 75°, 75°
Average −45° w	−15°, −15°, −45°, −45°, −75°, −75°
Average −45° w/o	−15°, −15°, −15°, −75°, −75°, −75°

The key difference between w and w/o is whether the set contained the average orientation itself or not.

In Experiment 2A, the participants’ task was to indicate with a button whether the average orientation of the set was tilted clockwise or counterclockwise relative to the vertical (Figure 1, center panel). Note that in both Experiments 1 and 2, we instructed participants not to attend to only a subset of the six patterns but to the entire display to ensure that participants did not explicitly change their strategy depending on the task. Moreover, the two EEG experiments (Experiments 1 and 2A) were conducted on the same day. These facts ensure the validity of our cross-decoding analysis (see below).

In Experiment 2B, participants were asked to report perceived average orientation by adjusting a white rotating bar presented at the center of the monitor (Figure 1, right panel) instead of a binary response. Note that participants were instructed to accurately report the average orientation they initially perceived and not to change it while rotating the bar. There was no response time limit; however, the mean response time was 3,627 ± 813 (mean ± SD) ms across participants, indicating that participants responded quickly based on our instruction, given the time needed to align the bar with their perceived average orientation. Also, we used four additional stimulus sets that served as dummy stimuli as well as the ones listed in Table 1: stimulus set to prevent participants from being aware that they were always presented with only a few stimulus sets and reporting almost the same orientation on all trials. The components of the dummy stimuli are as follows: (−5°, −5°, −35°, −35°, −65°, −65°), (5°, 5°, 35°, 35°, 65°, 65°), (30°, 30°, 45°, 45°, 60°, 60°), and (−30°, −30°, −45°, −45°, −60°, −60°). The trials in which the dummy stimuli were presented, or the sign of the response was opposite to that of the true average (e.g., a trial with a response of −20° when the set had an average of 45°) were excluded from the following analyses. Experiment 2B was conducted on an average of 21 days after the EEG experiments. Participants completed 4 blocks of 120 trials in Experiments 2A and 2B, except for one participant who only completed 2 blocks in Experiment 2A due to time constraints.

### 2.4 EEG recording and preprocessing

In Experiments 1 and 2A, the EEG signals were recorded at a sampling rate of 1,000 Hz from 32 electrodes (BrainVision Recorder, BrainAmp Amplifier, EasyCap, BrainProducts) located at FP1, FP2, F3, F4, F7, F8, Fz, T7, T8, C3, C4, Cz, FC1, FC2, FC5, FC6, P3, P4, P7, P8, Pz, TP9, TP10, CP1, CP2, CP5, CP6, PO3, PO4, O1, O2 and Oz. An electrode located between Fz and Cz served as a reference. The impedance of all electrodes was kept below 5 kΩ throughout the experiments.

We preprocessed the raw EEG signals with the EEGLAB toolbox for MATLAB. The raw EEG signals were downsampled to 500 Hz, band-pass filtered between 1 and 80 Hz, and epoched from 1,000 ms before to 1,500 ms after stimulus onset. The epochs were visually inspected to remove trials containing transient muscular activity and electrodes with persistent noise. Epochs with incorrect responses were also removed. Next, independent component analysis (ICA) was performed and the ICLabel plugin (Pion-Tonachini et al., 2019) was used to automatically obtain estimated labels for each component. Components with an estimated probability of more than 50% being artifacts (i.e., a label of eye, muscle, heart, line noise, or channel noise) were removed, resulting in an average of 21 ± 2.5 (mean ± SD) components across all participants and experiments. Epochs were baseline corrected with respect to the pre-stimulus period from −300 to 0 ms. This procedure resulted in 922 ± 136 (mean ± SD, Experiment 1) and 444 ± 150 (mean ± SD, Experiment 2A) trials for further analyses.

### 2.5 Decoding orientations with linear classification

**Figure 2:**
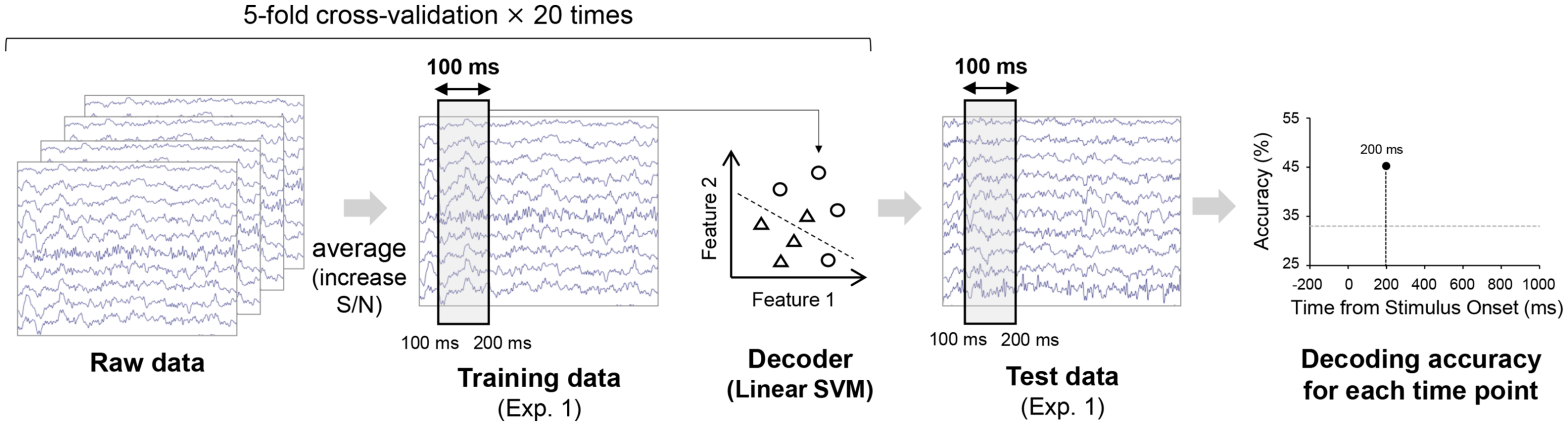
Procedure for the decoding analyses. The raw EEG data from Experiment 1 was divided into five sets for each orientation (i.e., 75°, 45°, 15° or −75°, −45°, −15°) and averaged within each set (up to 40 trials) to increase the signal-to-noise ratio. Using the four sets of averaged EEG patterns for each orientation within a 100 ms time window for each orientation, we trained a linear SVM classifier, which was tested on the held-out one set of averaged EEG patterns (one for each orientation) from Experiment 1 to obtain accuracy for decoding the 3-class orientation. Multiple decoders were constructed independently for each time window. The time course of decoding accuracy was obtained after repeating this procedure for the five cross-validation splits and with 20 different assignments of the raw EEG data to the five sets. All these analyses were performed individually for each participant.

**Figure 3:**
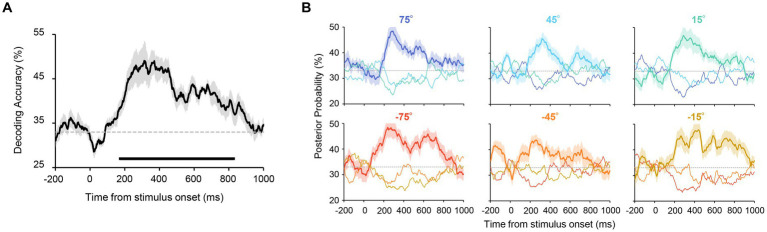
**(A)** Accuracy of orientation decoding. Orientation was reliably decoded from 174 to 828 ms after stimulus onset. The chance level (33%) is shown as a dashed line. The shaded area shows the standard error across participants. The horizontal line below the graph indicates time points that achieve significant decoding accuracy (revealed by a cluster-based permutation test with cluster-defining threshold *p* < 0.05 and cluster threshold *p* < 0.05). **(B)** Posterior probabilities of the presented orientations in Experiment 1. These values were computed by using the output of the decoders (see Methods for more details). Each panel shows the time course of the probability for each stimulus set with uniform orientations (shown above the panel). The solid lines correspond to the results for the presented orientation. The probabilities for the other two orientations (e.g., 45° and 15° when 75° was presented) are shown as thin lines. The probability of the presented orientation was higher for all stimulus sets after stimulus onset. This confirms that all presented orientations were reliably decoded.

To test if orientation could be decoded from EEG signals for our stimulus setting, orientation decoders were constructed in a time-resolved manner for each participant using potentials within a 100 ms time window and from ten occipital and parietal electrodes (P3, P4, P7, P8, Pz, PO3, PO4, O1, O2, Oz) recorded in Experiment 1 (Figure 2). These ten electrodes were selected in accordance with a previous study (Roberts et al., 2019). We trained two linear SVM classifiers (with a regularization parameter C of 1) for each participant to discriminate between three orientations included in each stimulus set of Experiment 2 (i.e., 75°/45°/15° classifier or −75°/−45°/−15° classifier). Specifically, the number of trials were first matched for each orientation in Experiment 1 through undersampling. The spatiotemporal EEG patterns for each orientation were then divided into 5 sets and the patterns within each set were averaged for each orientation to increase the signal-to-noise ratio, thus decoding accuracy (Grootswagers et al., 2017). Four sets were used as training data while the remaining set was used as test data. The averaged signal within each set was *z*-scored using the mean and standard deviation of the training data over −300 ms to 900 ms relative to stimulus onset, in accordance with the procedure used in a previous study (Hermann et al., 2022). Subsequently, a linear multiclass support vector machine (SVM) classifier was trained on the training data (12 samples = 4 sets × 3 orientations) and tested on the remaining 3 samples (1 set × 3 orientations). This procedure was repeated 5 times until all sets had served as test data (5-fold cross-validation). The entire procedure was repeated 20 times with samples randomly assigned to each set. This resulted in 100 decoding accuracies, averaged together to obtain a single decoding accuracy for a single time window. The 100 ms time window was shifted by a time step of 2 ms and the same training and testing procedure was performed, resulting in time courses of 3-class orientation decoding accuracy over −300 ms to 900 ms relative to stimulus onset (Figures 2, 3A).

We also computed three posterior probabilities of the three orientations (75°/45°/15° or −75°/−45°/−15°) from each decoder to confirm whether all six orientations were decodable. We defined SVM decision function values (obtained from sklearn.svm.LinearSVC.decision) passed through a softmax function as posterior probabilities for the test data. This procedure was again performed 100 times (5-fold cross-validation × 20 times) to generate 300 (=3 orientations × 100 repetitions) probabilities, which were averaged over 100 repetitions to obtain three posterior probabilities for each orientation. Time courses of posterior probabilities were obtained for three orientations by shifting the time window and repeating the same procedure. For further analyses, all 32 electrodes and different types of classifiers were used to construct decoders, and similar results were obtained (Supplementary Figures S1, S2).

### 2.6 Decoding ensemble representation with inverted encoding models

After we confirmed that EEG signals contained orientation information, we adopted a reasonable computational model to estimate when ensemble representations emerge during an ensemble judgment task based on previous studies (Oh et al., 2019). Specifically, we constructed inverted encoding models (Brouwer and Heeger, 2009, 2011; Sprague et al., 2015) to decode orientation representations at each time point.

**Figure 4:**
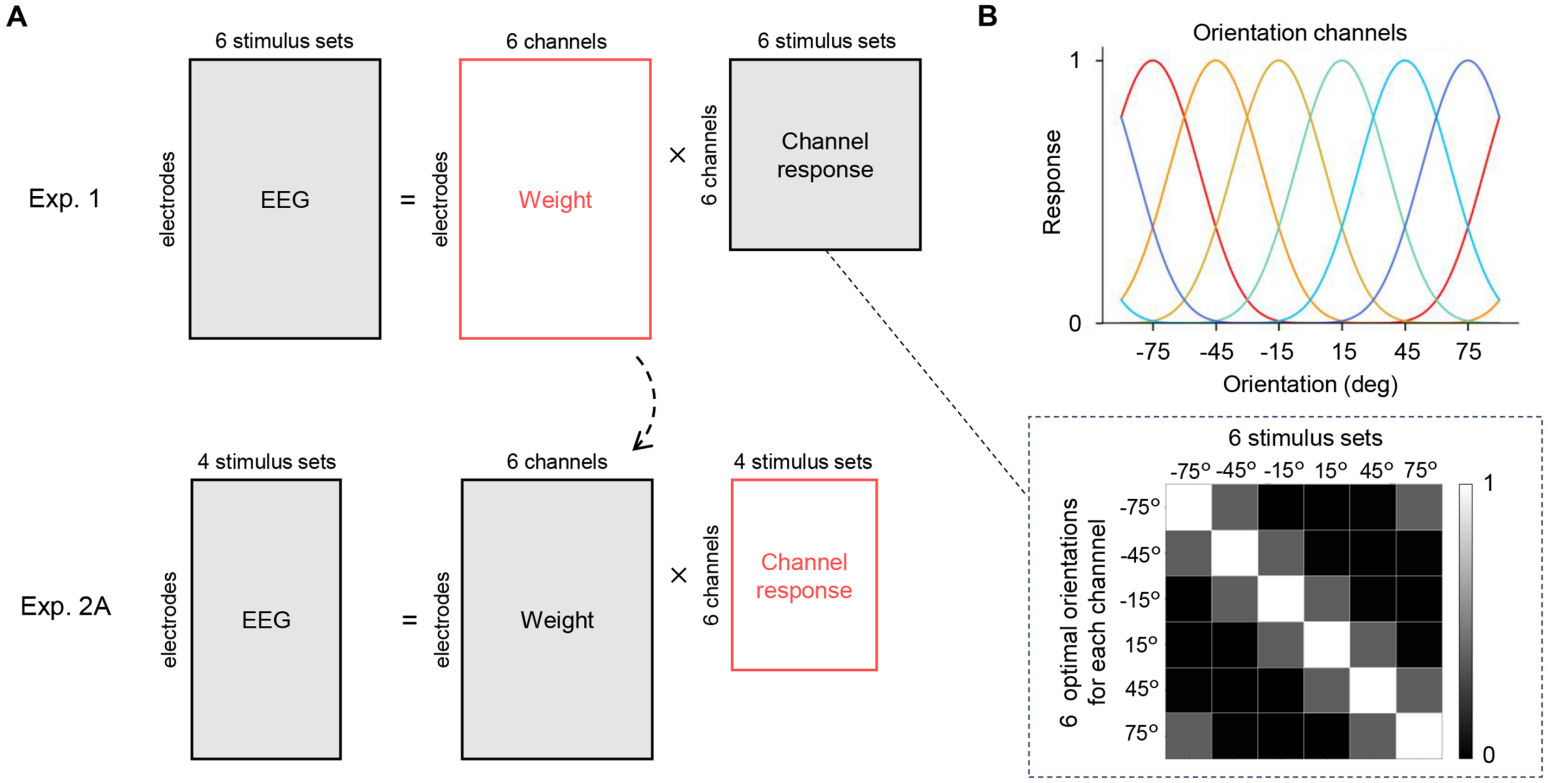
Inverted encoding model analysis. **(A)** The encoding model assumed that the EEG signals at a given time point can be modeled as a linear weighted sum of hypothetical orientation channel responses to the six orientations used in Experiment 1. The matrices to be estimated are shown in red. We first computed a least-squares estimate of the weight matrix (top row). Importantly, this matrix was applied to the EEG data in Experiment 2A (orientation ensemble discrimination task) to estimate the channel response matrix (bottom row), which corresponds to the representational strength of the six orientations while participants perceived an ensemble of the four stimulus sets in Experiment 2A (shown in Figure 5A). **(B)** The channel response matrix is illustrated as a grayscale map in the dashed box. Each column has response values of the channels to the stimulus set (single orientation). These response values are determined by the response functions of the channels (top) that were set based on the response property of orientation-selective neurons in the visual cortex.

This model is based on a biologically plausible assumption that EEG signals can be modeled as a linear weighted sum of population responses of multiple orientation-selective neurons in the brain. Following previous studies (Oh et al., 2019; Tark et al., 2021), we assumed an equal number of orientation channels as orientations used in the experiments, each tuned to a specific orientation (i.e., 75°, 45°, 15°, −75°, −45°, −15°). The response of each channel is described as:

(1) $$R=\left|\cos^{7}\left(\theta -\theta_{\text{max}}\right)\right|$$

where θ is an orientation presented to the channel and $\theta_{\text{max}}$ is an optimal orientation that maximizes the response of each channel. Next, we modeled EEG signals at each electrode as a linear weighted sum of the six hypothetical orientation channel responses. Let m be the number of electrodes, n be the number of experimental conditions (stimulus sets), and k be the number of hypothetical orientation channels (six, in our case). The matrix of EEG signals to each stimulus set $\left(B\in R^{m\times n}\right)$ is equal to the multiplication of a weight matrix $\left(W\in R^{m\times k}\right)$ and the matrix of hypothetical orientation channel responses $\left(C\in {\left[0,1\right]}^{k\times n}\right)$ (Figure 4A, top):

(2) B=WC

The response matrix C has response values (defined in Eq. 1) corresponding to the presented orientation in each column (Figure 4B). First, we estimated the weight matrix *W* using EEG data from Experiment 1 and the channel response matrix *C*. We then applied the estimated weight matrix to the EEG data from Experiment 2A to compute the channel response matrix for stimulus set w and w/o (Figure 4A bottom). This enabled us to investigate whether and when representations corresponding to the average orientation (i.e., 45°/−45°) emerge in the brain during orientation ensemble perception.

We performed 5-fold cross-validation as in the first decoding analysis to estimate the weight matrix W for our EEG data (see Decoding orientations with linear classification). The training data $B_{1}$ from Experiment 1 (EEG signals averaging within the 100 ms time window) and the response matrix $C_{1}$ were used to compute the least-squares estimate of a weight matrix:

(3) $$\hat{W}=B_{1}C_{1}^{T}{\left(C_{1}C_{1}^{T}\right)}^{-1}$$

The estimated weight matrix $\hat{W}$ was then applied to the test EEG data $B_{2}$ from Experiment 2A to estimate six orientation channel responses.

(4) $$\hat{C_{2}}={\left({\hat{W}}^{T}\hat{W}\right)}^{-1}{\hat{W}}^{T}B_{2}$$

This procedure was repeated 100 times with random data partition for each participant, and the estimated response matrices were averaged across all folds and repetitions. As the estimated response matrix reveals the response strength for each hypothetical channel to each stimulus set, we can use this matrix to quantify the representational strength of each orientation, especially the average orientation. Repeating this procedure for all time points yields the time courses of the representational strength of each orientation for each stimulus set.

To quantify the strength of representations corresponding to the average orientation, we extracted a representative value, referred to as orientation sensitivity, from the estimated responses of each channel, following the approach adopted in previous studies (Oh et al., 2019; Sutterer et al., 2019). Specifically, we first circularly shifted the responses of orientation channels along the rows such that the responses of the channel tuned to the average orientation aligned at the center (fourth row of the matrix). Subsequently, we averaged the response of orientation channels along the columns (within the stimulus set), which yielded two vectors of six elements for each stimulus set type (w and w/o). Each vector had the central (fourth) value representing the strength of the representation corresponding to the average orientation of the stimulus set and the adjacent values representing the strength of representations corresponding to orientations ±30°, ±60°, and −90° away from the average orientation. Furthermore, we performed linear regression on these six channel response values, disregarding the sign of orientation (fitting a line to the response values for −90°, −60°, −60°, −30°, −30°, 0°). We defined the slope of the fitted line as orientation sensitivity. Positive orientation sensitivity indicates the presence of a peak in the channel responses, suggesting strong representations corresponding to the average orientation for Experiment 2; in contrast, zero sensitivity indicates no representations corresponding to the average orientation.

### 2.7 Estimating the timing of full formation of orientation ensemble representation corresponding to the behavior

To gain further insights into when orientation representations corresponding to the participants’ perceived average orientation emerge in the brain, we used the estimated channel responses to decode orientation continuously at each time point using the same approach as a previous study (Brouwer and Heeger, 2009). This analysis included computing six channel responses ( R in Eq. 1) to 180 orientations ranging from −89° to 90°, correlating these responses with the estimated responses in the cross-decoding analysis, and choosing the orientation that showed the highest correlation among those with the same sign as the average orientation of the stimulus set. Subsequently, these decoded orientations were tested to assess the correlation with participants’ perceived average orientations in Experiment 2B. A higher correlation at one time point makes it more likely that the ensemble perception was fully formed in the brain at that time point. Note that the correlations for stimulus sets w and w/o were calculated by collapsing the data over the true average orientation (45° or −45°). Specifically, the true average orientation was subtracted from both the estimated orientation and the perceived average orientation. Then, the correlation between these two values was computed for each time point, resulting in two-time courses of correlation for stimulus sets w and w/o.

### 2.8 Statistical tests

We assessed the statistical significance of orientation decoding accuracy using a cluster-based permutation test. The orientation labels for the EEG data were permuted and the orientation decoding accuracy was recalculated according to the procedure described above. After repeating this procedure 1,000 times, null distributions of decoding accuracy were generated for each time window, from which the *p*-values for the actual decoding accuracy were calculated. The original decoding accuracy curve was thus transformed into a time course of *p*-values. Clusters were defined as neighboring time points at which all *p*-values were below a significance level, and deemed significant if their size exceeded the calculated threshold from a null distribution of cluster size. The same procedure was used to assess the statistical significance of sensitivity in the estimated orientation channel responses.

Regarding the correlation coefficients between the perceived average orientation and the estimated orientation representation, we again permuted the orientation labels for the EEG data and constructed inverted encoding models to estimate the weight matrices for each participant. These matrices allowed us to obtain orientation channel responses and estimated orientation at each time window as described above. We computed the correlation coefficient between the estimated orientations and perceived average orientations that participants reported. Repeating this procedure 1,000 times resulted in null distributions of the correlation coefficients for each time window. We again performed a cluster-based permutation test to assess the statistical significance of the observed correlation coefficients for each stimulus set.

## 3 Results

We aimed to estimate the time when accurate ensemble representations were formed in the brain. To this end, we conducted EEG recordings and psychophysical experiments to decode the dynamics in the representational strength of multiple orientations while human observers judged their average orientation. Figure 1 shows an overview of our psychophysical experiments. Eleven adults participated in all experiments in which six Gabor patterns were presented around a fixation point for 500 ms. We recorded EEG signals while participants discriminated Gabor patterns comprising uniform orientations (Experiment 1) and discriminated the average orientation of Gabor patterns with different orientations (Experiment 2A). We also asked participants to report the perceived average orientation on a continuous scale by rotating a bar in another experiment (Experiment 2B).

### 3.1 Orientation decoding

We first tested whether the uniform orientation of six Gabor patterns could be reliably decoded from EEG signals. Specifically, orientation decoders were trained and tested using EEG signals (from ten occipital and parietal electrodes) elicited by six Gabor patterns with identical orientations (Figure 1, left panel). Participants successfully discriminated the orientation of the patterns (clockwise or counterclockwise inclined relative to the vertical) in most trials with a mean accuracy of 98.4% (SD = 0.02%). An orientation decoder was constructed using EEG signals within a 100 ms time window that was shifted in 2 ms steps, resulting in a time course of orientation decoding accuracy (Figure 2). Note that two types of decoders were constructed, each discriminating orientations contained in the stimulus set used in Experiment 2 (i.e., 75°/45°/15° decoders or −75°/−45°/−15° decoders). Figure 3A shows that decoding accuracy reached significance at 174 ms and remained above chance until 828 ms after stimulus onset (cluster-based permutation test; *p* < 0.05 cluster-defining threshold; *p* < 0.05 cluster-threshold).

To test whether all six orientations were reliably decodable, the posterior probabilities of each orientation were calculated for each stimulus set by using the output of the decoder (see Methods). As can be seen in Figure 3B, for each stimulus set, we obtained the highest probability for the presented orientation throughout the stimulus presentation (e.g., the probability of 75° was higher than that of 45° and 15° when 75° was presented), ensuring that all six orientations were reliably decoded.

### 3.2 Decoding ensemble representations with inverted encoding models

The results of Experiment 1 showed that the orientation information was reliably decoded from the EEG signals. Next, we constructed an encoding model that predicts EEG signals by assuming biologically plausible orientation channels to examine when ensemble representation is formed in the brain during ensemble perception. This model was then inverted to decode the temporal evolution of the representational strength of multiple orientations (inverted encoding models) (Brouwer and Heeger, 2009, 2011; Sprague et al., 2015; Oh et al., 2019; Sutterer et al., 2019) from EEG signals in Experiment 2. In this experiment, we utilized six Gabor patterns with two or three different orientations and an average of 45° or −45° (Table 1). For each average orientation, two stimulus sets, with and without elements corresponding to the average (w and w/o), were created to see whether the temporal dynamics of the representational strength of each orientation were affected by the presence of the average orientation pattern in the set. Participants were asked to indicate whether the average orientation was tilted clockwise or counterclockwise relative to the vertical with a binary response.

The critical idea of inverted encoding models is to assume multiple orientation channels (derived from populations of multiple orientation-selective neurons in the brain) and model EEG signals at each electrode and time point as a linear weighted sum of the channel responses (Figure 4B). This analysis is explained in detail in the Methods and briefly summarized as follows: we first divided the EEG data into training and test datasets, consistent with our initial decoding analysis. We then estimated a weight matrix for six hypothetical orientation channels corresponding to the six orientations used in Experiment 1 (Figure 4A, top). This weight matrix was subsequently applied to the EEG data from Experiment 2A to estimate the responses of the hypothetical orientation channels during ensemble perception (Figure 4A, bottom). Importantly, these responses can be considered as the representational strength of each orientation at a given time point. We repeated this procedure for all time points and participants to investigate how orientation representation evolves over time during orientation ensemble perception. We hypothesized that if ensemble representation is explicitly formed in the brain at a specific time point, a significantly high response of the hypothetical orientation channel tuned to the average orientation (45° or −45°) would be observed at that time, allowing us to estimate the exact time the ensemble perception was formed.

**Figure 5:**
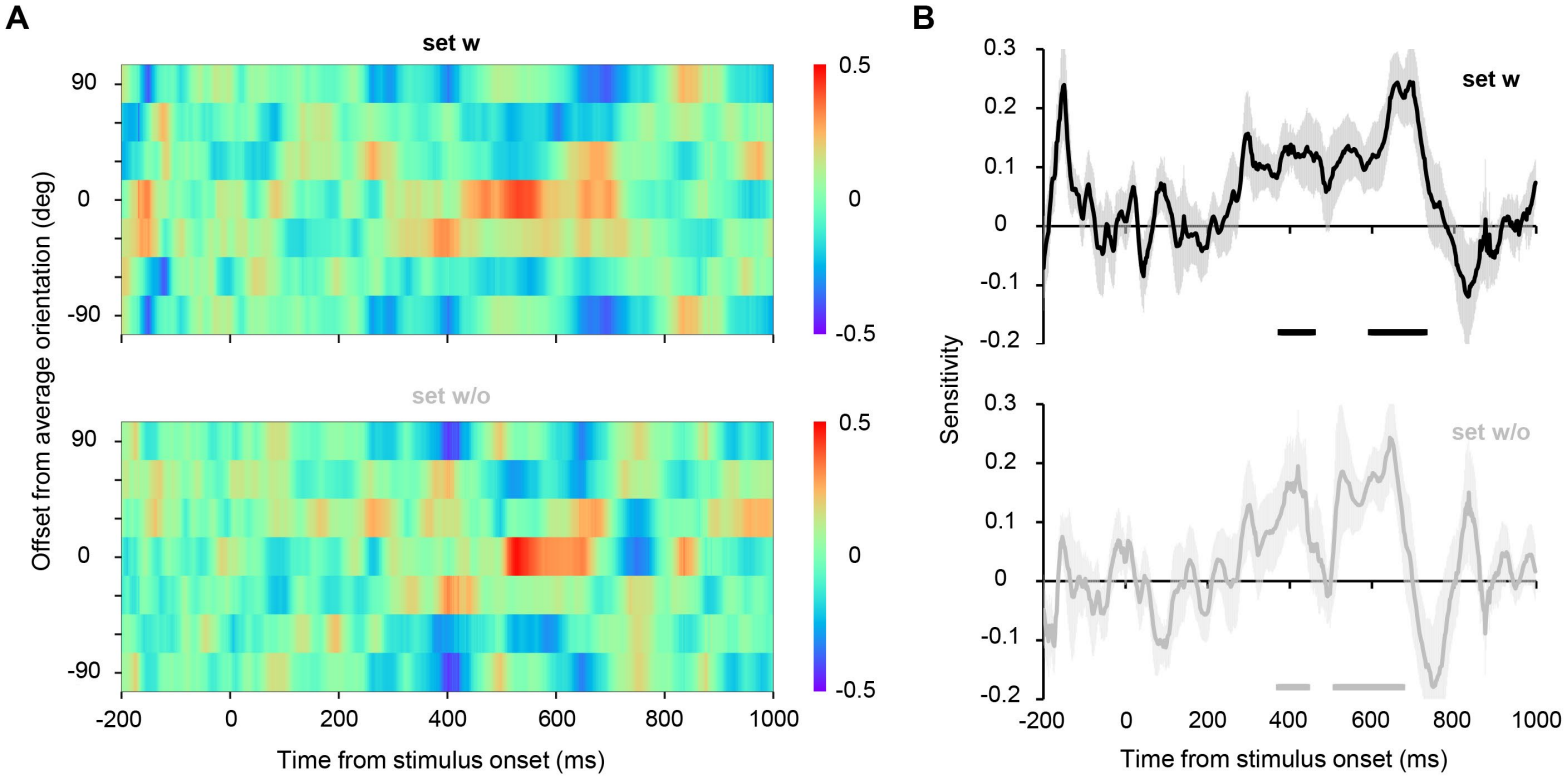
**(A)** Estimated channel responses in the inverted encoding models over time for Experiment 2A. The channel responses were circularly shifted such that the channel response to the average orientation of each set aligned at the center in the ordinate. For illustrative purposes, we have duplicated the top row of the color map and added it as the seventh row. The red center cells and the blue neighboring cells at approximately 400–700 ms indicate a clear peak in the channel response profile; that is, the representational strength of the average orientation of the stimulus set was higher than that of the other orientations. **(B)** A value referred to as sensitivity was computed from the channel response profile at each time point to quantitatively assess the representational strength of the average orientation relative to the other orientations. The shaded area shows the standard error across participants. The horizontal lines below the plots indicate time points that achieve significant sensitivity (significantly higher representational strength of the average orientation), revealed by a permutation test with cluster-defining threshold *p* < 0.05 and cluster threshold *p* < 0.05.

We estimated the hypothetical orientation channel responses for the EEG data from Experiment 2A, wherein EEG signals were recorded while participants discriminated the average orientation of the set (2AFC discrimination task, Figure 1, right panel; behavioral performance averaged 92.6%, SD = 0.10%). Note that participants were instructed to attend to the entire display during the task to minimize the possibility that they merely attended to only one element to achieve such high accuracy. The color maps in Figure 5A show the estimated responses of hypothetical orientation channels for each time point. The central row of the map represents the responses of the channel tuned to the true average orientation (45°, −45°), indicating the representational strength of the true average orientation in EEG signals. We found that for both sets w and w/o, the representation of the true average orientation was high between 400 and 700 ms after stimulus onset. To quantitatively evaluate the strength of the representation of the true average orientation (the degree of the peak in the channel response profile), we collapsed the response values across the signs of the orientations and fitted a line to them to estimate its slope, which we refer to as orientation sensitivity (Oh et al., 2019; Sutterer et al., 2019). Figure 5B shows that significantly high orientation sensitivity was observed in 376–474 ms and 592–732 ms for set w, and 374–442 ms and 512–672 ms for set w/o, indicating the emergence of ensemble representation at later time points. These findings reveal that ensemble representation emerges at 400–500 ms during the perception of orientation ensembles and is sustained till approximately 700 ms after stimulus onset, regardless of the presence of the average orientation in the stimulus set.

### 3.3 Estimating the timing of orientation ensemble representation corresponding to the behavior

**Figure 6:**
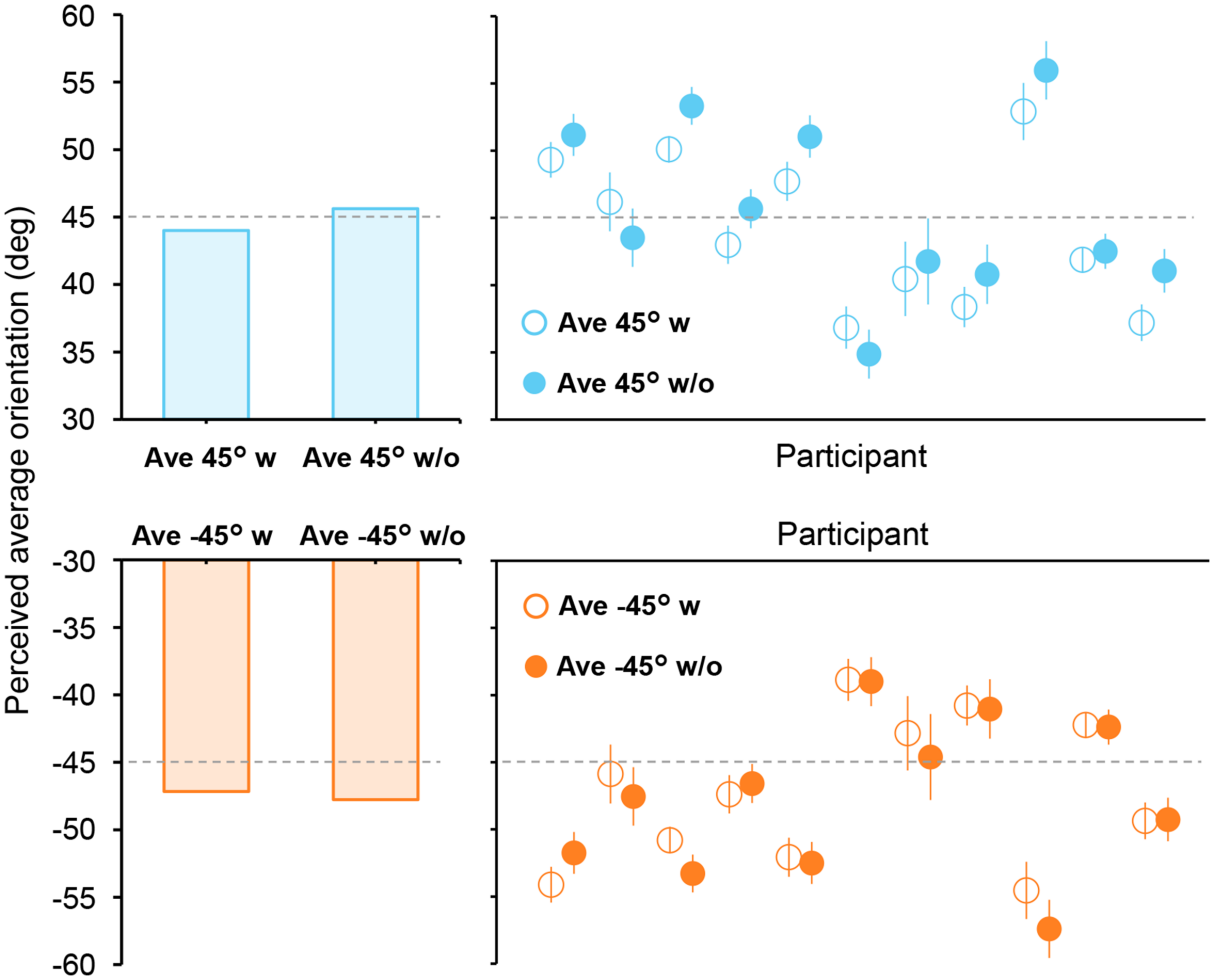
Perceived average orientations in Experiment 2B. The left bars show the mean perceived orientation of the participants. Each circle in the right panels represents the perceived orientation for each participant. The error bars indicate the standard error across all trials. The true average is shown as a dashed line. Most participants perceived the average orientation correctly, with errors mostly within ±10°.

To more precisely estimate the time at which ensemble representation is fully formed in the brain, we computed the neural correlate of the participants’ perceived average orientation. This is based on our idea that a high correlation between the decoded orientation representation and the perceived average orientation at a specific time point indicates the complete formation of ensemble representation corresponding to an individual’s perception. Therefore, to precisely measure the average orientation perceived by each participant, we conducted another psychophysical experiment using adjustment methods in which participants indicated the perceived average orientation on a continuous scale by rotating a bar stimulus (Experiment 2B; Figure 1, right panel). Figure 6 shows the perceived average orientations for each participant and stimulus set. The perceived orientations varied from participant to participant; however, they were mostly close to the true average, indicating that participants perceived the average orientation quite well.

**Figure 7:**
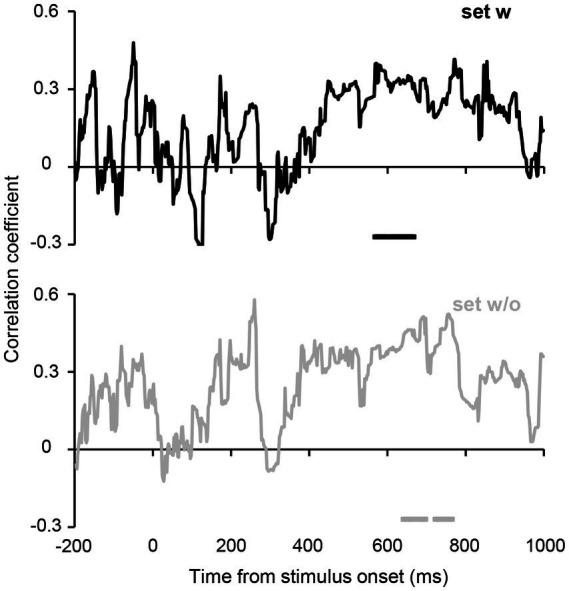
Correlations between the perceived average orientation in Experiment 2B and orientation decoded from the estimated orientation channel responses. The horizontal lines below the plots indicate time points that achieve significant correlation. Although the time courses show transient fluctuations (presumably due to the relatively small sample size), a cluster-based permutation test revealed that correlations remained significant in the time range of 568–668 ms for set w, and 640–698 and 722–766 ms for set w/o (cluster-defining threshold *p* < 0.1 and cluster threshold *p* < 0.1), suggesting that orientation ensemble representation corresponding to the individual ensemble perception emerged over 600–700 ms after stimulus onset.

Furthermore, we computed the correlation between the participants’ average orientations in Experiment 2B and orientation decoded from the estimated hypothetical channel responses at each time point (see Methods for further details). Figure 7 shows the time courses of the correlations. Despite unstable correlation coefficients observed in the earlier period, which are likely due to the relatively small number of participants, a cluster-based permutation test revealed significant correlations at 568–668 ms for set w and slightly later time points (640–698 and 722–766 ms) for set w/o. These results suggest that ensemble representation corresponding to the individual perception is fully formed at 600–700 ms after stimulus onset.

## 4 Discussion

We investigated the temporal dynamics of orientation ensemble perception using psychophysical experiments and time-resolved orientation decoding analysis from EEG signals. One of the keys was using inverted encoding models for each time point across the experiments (single and ensemble orientation discrimination task; Experiment 1 and 2A) to quantify the representational strength of individual and ensemble orientations. Another key was that the neural orientation representations predicted from the inverted encoding models were directly correlated with the perceived average orientations in the task with adjustment methods. The time course of the correlation allowed us to accurately estimate when ensemble representations associated with individual perception are fully formed in the brain, which would otherwise not have been possible for behavioral paradigms with discrete responses used in most studies of ensemble perception (member identification task or 2AFC task).

The time courses of the sensitivity of the channel response profile (Figure 5B) have shown that the representation of orientation ensembles emerges at approximately 400 ms and remains strong until approximately 700 ms. Additionally, our correlation analysis (Figure 7) has revealed significant correlations between the perceived average orientation and the orientation decoded from the estimated channel response at approximately 600–700 ms after stimulus onset. Taken together, ensemble perception could be formed over several hundred milliseconds. However, previous behavioral studies have suggested a much shorter timescale (50–200 ms) for computing ensembles based on the invariant performance of ensemble discrimination to stimulus duration (Chong and Treisman, 2003; Robitaille and Harris, 2011; Yamanashi Leib et al., 2016). These results can be reconciled as follows. A crucial problem with previous behavioral studies (Chong and Treisman, 2003; Robitaille and Harris, 2011) is that they used unmasked visual stimuli that allowed observers to access stimulus information beyond the actual stimulus duration, potentially affecting behavioral performance. Indeed, some studies using backward masking have shown that ensemble perception performance improves with a stimulus duration of 50 to 500 ms (Epstein et al., 2020). Therefore, it is reasonable to assume that the brain does not fully form an ensemble representation within 50–200 ms, which may explain the absence of the orientation ensemble (45°/−45°) representation in the early periods of our data. Moreover, reaction times for ensemble judgments are typically 600–1,000 ms (Robitaille and Harris, 2011; Li et al., 2016), consistent with the present results given the delay associated with the motor response. Considering these facts, ensembles can be roughly extracted within 50–200 ms; however, the visual system could gradually form ensemble perception over several hundred milliseconds to achieve a more accurate ensemble representation.

Despite this seemingly reasonable conclusion, there is still a small but critical discrepancy between our results and previous neuroimaging studies regarding the timing of ensemble representation. One EEG study employed working memory tasks in which human participants recalled the orientation of a cued stimulus among multiple stimuli. Although participants were not explicitly required to perceive an ensemble of these stimuli, inverted encoding models decoded ensemble representations at approximately 300 ms after stimulus onset (Oh et al., 2019), slightly earlier than our findings suggest (400 ms). We speculate that this difference in the timing of ensemble representations can be attributed to the variation in task types and stimulus settings. The previous study placed an array of orientations closer to the fovea, whereas orientations were more peripherally presented in our study. Consequently, ensemble representations emerged later in our study, as visual information is processed more slowly with increasing distance from the fovea (Lueschow et al., 1994; Carrasco et al., 1995). The stimulus duration also differed between these two studies (100 ms and 500 ms in the previous study and our study, respectively), which may allow participants in our study to spend more time sampling orientation information to form an orientation ensemble representation. Therefore, future studies need to use stimuli with various settings and from different domains, such as color, size, and facial expression, to gain a more comprehensive understanding of the temporal dynamics of ensemble perception.

Although inverted encoding models build upon biologically plausible assumptions given the response-tuning function of feature-selective neurons in the brain (Brouwer and Heeger, 2009, 2011; Sprague et al., 2015), there are several caveats for interpreting the results. Previous studies have shown that inverted encoding models are suitable for understanding population-level stimulus representation but not for understanding single-unit response property (Sprague et al., 2018). Therefore, our results do not necessarily mean that neurons tuned to the average orientation (45°/−45° in our case) are firing while humans perceive an ensemble of multiple orientations. However, the results revealed by our inverted encoding model are still noteworthy because we aimed to estimate the temporal evolution of the overall representation of individual and ensemble orientations in a multivariate pattern of EEG signals. The estimated channel responses (Figure 5) indicate that perceiving physically presented orientations of 45°/−45° shares common neural representations with perceiving an ensemble of orientations whose average is 45°/−45°, especially at later time points.

Another concern regarding the use of inverted encoding models is that what can be obtained from these models is the response of multiple channels, which experimenters arbitrarily choose from infinite possibilities, rather than a stimulus itself presented in an experiment (Gardner and Liu, 2019; Sprague et al., 2019). This led us to wonder if the decoded ensemble representation is spuriously observed as a natural consequence of the model irrespective of signals in EEG data. Notably, our additional analysis without arbitrary assumptions of channel response (where we simply applied the linear SVM classifiers in Experiment 1 to EEG data in Experiment 2A to obtain a posterior probability of each orientation as a measure of the representational strength of orientations) did not show significantly strong ensemble representations at any time point (Supplementary Figure S3). Nevertheless, we argue that our results from inverted encoding models are valid because we ensured through cluster-based permutation tests that if EEG data were associated with random orientation labels, the strong representation of the true average would not be observed even under the same model assumption. More specifically, the expected value of the null distribution of orientation sensitivity in the permutation test was nearly zero (no bias) for all time points. Therefore, this fact rules out the possibility that the observed ensemble representation is an artifact due to systematic biases introduced by the inverted encoding model (in Supplementary material, we provide additional discussion regarding why the different pattern of results was observed between the two analyses).

As stimulus displays varied across experiments (uniform and non-uniform orientations), they might have been processed differently by participants, potentially affecting the validity of using the same inverted encoding model across the tasks (cross-decoding analysis). For instance, participants may have attended to one orientation at a specific location for uniform orientations in Experiment 1, whereas they attended to the whole stimulus display in Experiment 2A. Although we cannot preclude the possibility that this difference in strategy affected our results, we argue that this possibility is minimized because we explicitly instructed participants to attend all orientations in Experiments 1 and 2. However, one might still argue that conducting decoding analysis within each experiment is preferable to cross-decoding. We had originally considered performing a simple classification of different stimulus sets within Experiment 2A, similar to previous studies (Roberts et al., 2019; Epstein and Emmanouil, 2021). However, such a binary classification would not be sufficient to distinguish between ensemble and non-ensemble processing. Specifically, processing a subset of elements instead of forming an ensemble representation from all the elements could potentially lead to significant classification accuracy for the two sets. Therefore, we argue that conducting cross-decoding analysis and correlating the estimated orientation representations with perceived average orientations are essential to determining the exact timing of ensemble perception.

Two main factors potentially affect our results: the low signal-to-noise ratio in EEG data and a relatively small number of participants for the correlation analysis. These factors may have spuriously delayed the decoding of ensemble representation even though the brain represents an ensemble at earlier time points. However, we employed some strategies and rigorous statistical tests to minimize biases introduced by these factors to our results. These strategies included averaging the EEG data across several trials and using multiple data points within each time window as features for the decoder to maximize the signal-to-noise ratio (Grootswagers et al., 2017). Regarding the correlation analysis with a relatively small number of participants, we performed a cluster-based permutation test to rigorously assess the significance of correlation coefficients, given the inherent temporal dependencies in EEG data. The timing of the high correlation is consistent with that of the highest response of the channel tuned to the average orientation of approximately 500–700 ms (Figures 5, 7). Therefore, these facts support the finding that ensemble representation corresponding to the individual perception emerges several hundred milliseconds after stimulus onset. However, further studies with other neural recording techniques will be necessary to conclude the robustness of our results. For instance, similar MEG experiments and analyses, which capture the similar neural representations to EEG but uniquely contain information represented in lower visual cortices (Cichy and Pantazis, 2017), would effectively corroborate the gradual emergence of accurate ensemble representation associated with ensemble perception.

Overall, our results are consistent with the notion of slower computation of orientation ensembles for a refined ensemble representation, although ensembles can be computed quickly and coarsely. By aligning our results with findings from previous studies (Myczek and Simons, 2008; Solomon et al., 2011; Allik et al., 2013), we infer computational mechanisms of ensemble perception: serial and focused-attention processing might underlie slow ensemble computation. However, this does not completely exclude the possibility that parallel processing is also involved in the computation of an ensemble. A combination of parallel and serial processing may underlie ensemble perception (Whitney and Yamanashi Leib, 2018; Baek and Chong, 2020). For example, some elements may be processed in parallel, with unequal weights assigned to each element, which may occur serially across different sets of elements over time. Alternatively, a single element can be sampled serially, followed by a one-off ensemble computation. Further studies will be necessary to investigate these possibilities and to describe the computational mechanisms of ensemble perception in more detail. A comparison of fMRI and EEG/MEG using dissimilarity matrices (Cichy et al., 2014; Hebart et al., 2018) could take advantage of high spatial and temporal resolution, contributing to a deeper understanding of how and when ensemble representations are formed in the brain. Also, since covert attention directed to specific elements or distributed across space is inherently involved in ensemble extraction (Chong and Treisman, 2005; Emmanouil and Treisman, 2008; Brand et al., 2012), decoding covertly attended visual stimuli (Noah et al., 2020) will facilitate further investigation of the temporal dynamics and computational mechanisms of ensemble perception.

## Data availability statement

The raw data supporting the conclusions of this article will be made available by the authors upon reasonable request.

## Ethics statement

The studies involving humans were approved by the Ethics Committee of the University of Tokyo. The studies were conducted in accordance with the local legislation and institutional requirements. The participants provided their written informed consent to participate in this study.

## Author contributions

RY: Conceptualization, Formal analysis, Funding acquisition, Investigation, Visualization, Writing – original draft, Writing – review & editing. MS: Supervision, Writing – review & editing. KA: Supervision, Writing – review & editing.
