# Supplementary material for: Decoding time-resolved neural representations of orientation ensemble perception
Source: Front Neurosci. 2024 Aug 1;18:1387393. doi: 10.3389/fnins.2024.1387393 (PMC11325722; doi:10.3389/fnins.2024.1387393)
Supplement: Supplementary file 1 [file Data_Sheet_1.docx]

Supplementary Material

Decoding time-resolved neural representations of orientation ensemble perception

**Ryuto Yashiro*, Masataka Sawayama, Kaoru Amano**

*** Correspondence:** Ryuto Yashiro: ryuto.yashiro@gmail.com

# Supplementary text: decoding ensemble representation with multi-class SVM

**1.1 Methods**

After we computed single orientation decoding accuracy in Experiment 1 using three-class SVM classifiers, we also used these classifiers to conduct a cross-decoding analysis to track the fluctuation in orientation representation while participants performed an ensemble perception task. This analysis involved testing the classifier for a single time window in Experiment 1 on the temporally corresponding EEG signals from Experiment 2A. The EEG signals from Experiment 2A were averaged over 30 trials (almost equal to the number of trials included in each of the five sets when training the decoders), and the z-score was obtained as stated in the main text. Furthermore, three posterior probabilities were obtained for the orientations (75°/45°/15° or -75°/-45°/-15°), which can be considered as the transient representational strength of each orientation, similar to a previous study (Rich & Wallis, 2016), following the definition and procedure in the main text (see Decoding orientations with linear classification; the results in Figure 3B confirmed that the probability computed from the output of the decoders truly reflects the processing of the presented orientation). Based on these probabilities, it was hypothesized that if the ensemble representation is explicitly formed at a specific time point, a significantly high probability of the true average orientation (45° or -45°) would be observed at that time, enabling us to estimate the exact time of formation of the ensemble perception.

**1.2 Results**

The time courses of the probabilities are shown in Supplementary Figure S3. Contrary to our hypothesis, for the set w/o (with no 45° or -45° pattern), the probabilities of the true average were not significantly higher than those of the other orientations throughout the period. Remarkably, this was also true for the set w, which contained the true average orientation as a local element. We also obtained qualitatively similar results when we constructed decoders with all electrodes (including the frontal and central electrodes as well as the original ten occipital and parietal electrodes) and different classifier types (Supplementary Figure S4).

The probabilities were mostly close to the chance level (33%) due to the large individual differences in the temporal profiles between participants (Supplementary Figure S5); however, we observed significantly higher probabilities of 75°/-75° at the group level. These high probabilities persisted until approximately 300 ms post-stimulus for two of the four stimulus sets (average 45° w/o and average -45° w/o) and in the later period for one stimulus set (average -45° w; cluster-based permutation test; *p* < 0.1 cluster-defining threshold; *p* < 0.1 cluster-threshold). Since 75° and -75° are most informative for the task goal, the visual system may selectively process task-relevant information, especially at an early stage of ensemble processing.

**1.3 Discussion**

There was no strong representation of the true average in the result above. However, we observed it in our analysis using inverted encoding models. We attribute the difference across these decoding analyses to the difference in orientation labels assigned to our EEG data. In our first decoding analysis with multi-class SVM, we used “hard” labels (i.e., one-hot vectors encoding the presented orientation itself) for our EEG data; in contrast, assuming broadly tuned channel responses in the inverted encoding model, where each takes a continuous value from 0 to 1, may be analogous to assigning “soft” labels to our EEG data associated with each stimulus set. We argue that such a biologically plausible assumption in the model may have facilitated the decoding of ensemble orientation weakly represented in the EEG signals.

An unequal representational strength of multiple orientations was found early, as shown by the higher posterior probability of 75° and -75° (Supplementary Figure S3). This suggests that the visual system may selectively process individual task-relevant elements, especially at an early stage of ensemble processing. The notion of selective processing can explain the fact that we observed the higher posterior probability of 75°/-75° only in the w/o sets: the w/o sets contained more 75°/-75° elements; therefore, it is more likely for 75°/-75° elements in the w/o sets to be selectively processed compared with those in the w sets. Moreover, the notion of selective processing is supported by a recent fMRI study showing a transient change in the BOLD signal in response to a few outliers in a collection of objects (Cant & Xu, 2020) and by behavioral studies showing that humans unequally weight visual elements distributed across space to make judgments about their ensemble (de Gardelle & Summerfield, 2011; Epstein et al., 2020; Haberman & Whitney, 2010; Lau & Brady, 2018; Li et al., 2017; Pascucci et al., 2021; Tiurina et al., 2024). Consistent with these findings, our study may provide complementary evidence for selective processing underlying ensemble perception regarding the relative strength of the neural representations of multiple elements.

This result could be influenced by a specific strategy that participants developed based on the stimulus setting with few orientations: despite the instruction to attend to the entire display, participants only focused on a highly tilted orientation to report the average orientation in Experiment 2A, as all orientations were on the same side of the vertical. This strategy could explain the significantly higher posterior probability of 75° and -75° (i.e., the most tilted orientations). Subsequent studies should investigate the temporal dynamics of orientation representations using patterns with more varied orientations. However, it might be technically difficult to reliably decode different orientations from EEG signals, especially those with a small (<30°) orientation difference (Cichy et al., 2015; Pantazis et al., 2018), reason for our decision to use the display with only a few orientations.

**2 Supplementary Figures**


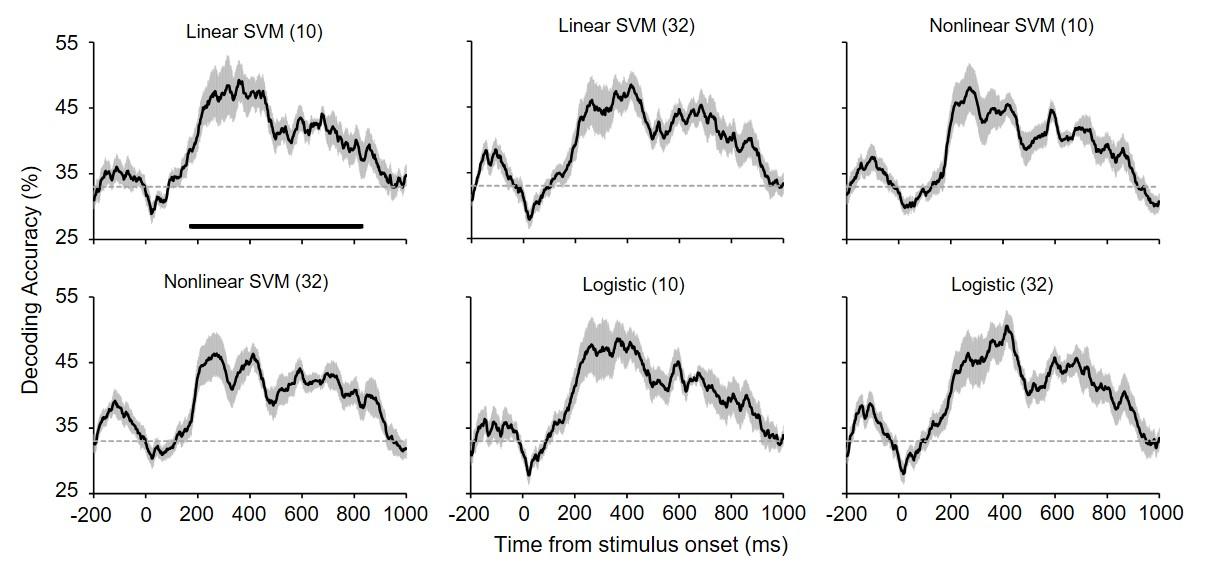


**Supplementary Figure S1.** Orientation decoding accuracy for different decoder types (linear SVM, nonlinear SVM or logistic regression) and the number of electrodes used to construct the decoders (in parentheses: 10 or 32). The upper left panel is the same as in Fig. 3A. The decoding accuracy profiles remained qualitatively similar, even though different types of decoders and all 32 electrodes were used. All other conventions are the same as in Fig. 3A.


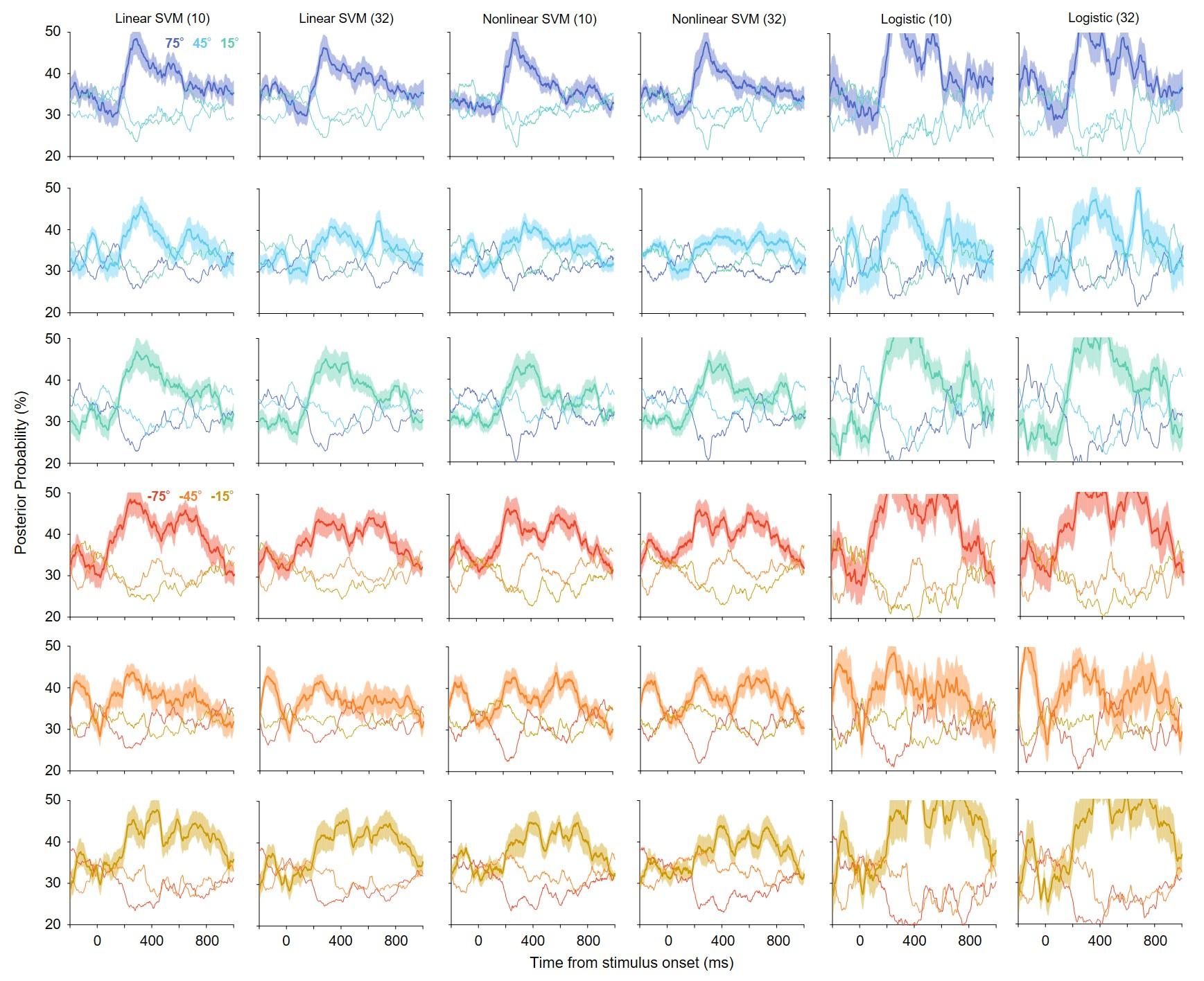


**Supplementary Figure S2.** Posterior probabilities of six orientations in Experiment 1 for different decoder types and the number of electrodes used to construct the decoders (in parentheses: 10 or 32). The panels in the left column are the same as in Fig. 3B. The probability profiles were qualitatively similar for the different types of decoders and electrodes. All other conventions are the same as in Fig. 3B.


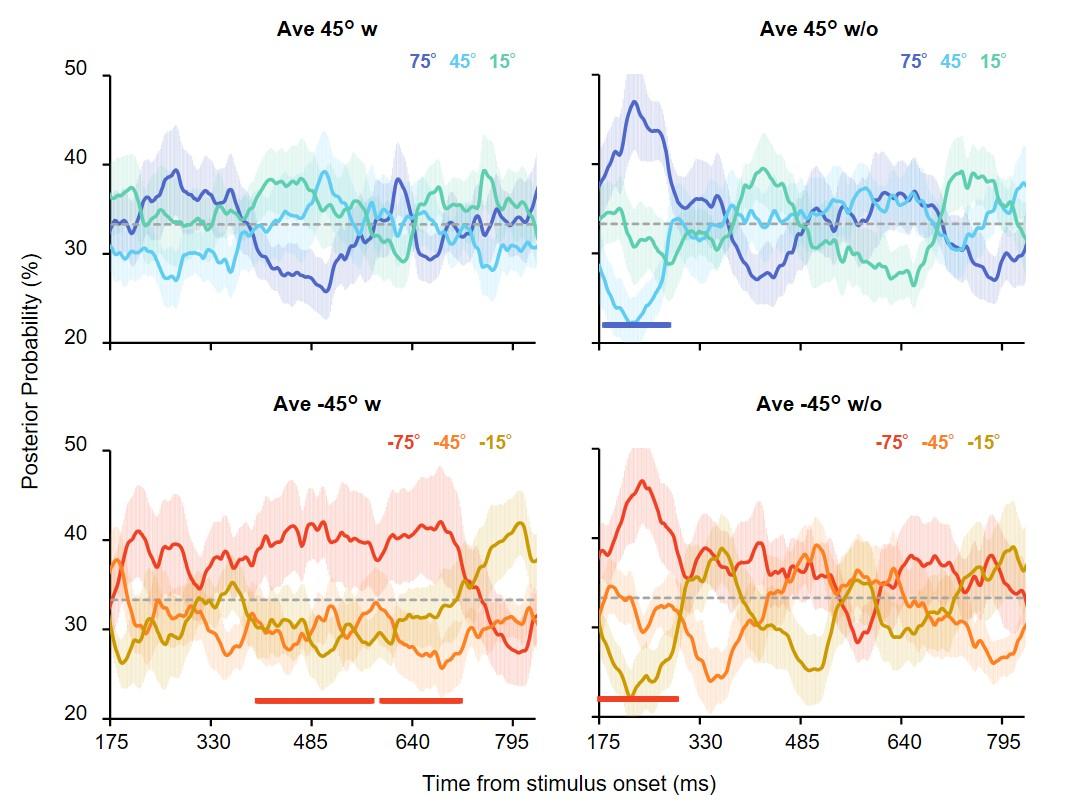


**Supplementary Figure S3.** Posterior probabilities of the orientations that constituted the stimulus sets in Experiment 2A. Each panel shows the results for each stimulus set. Each color represents a specific orientation. The time range is restricted to the period in which decoding accuracy above chance was achieved (Figure 3A). The shaded area shows the standard error across participants. The probabilities of the true average (45° and -45°, shown as light blue and orange lines, respectively) were not high across all time points. In addition, significantly high probabilities were observed for individual orientations (75° or -75°), indicated by the horizontal lines below (permutation tests with cluster-defining threshold *p* < 0.1; cluster threshold *p* < 0.1).


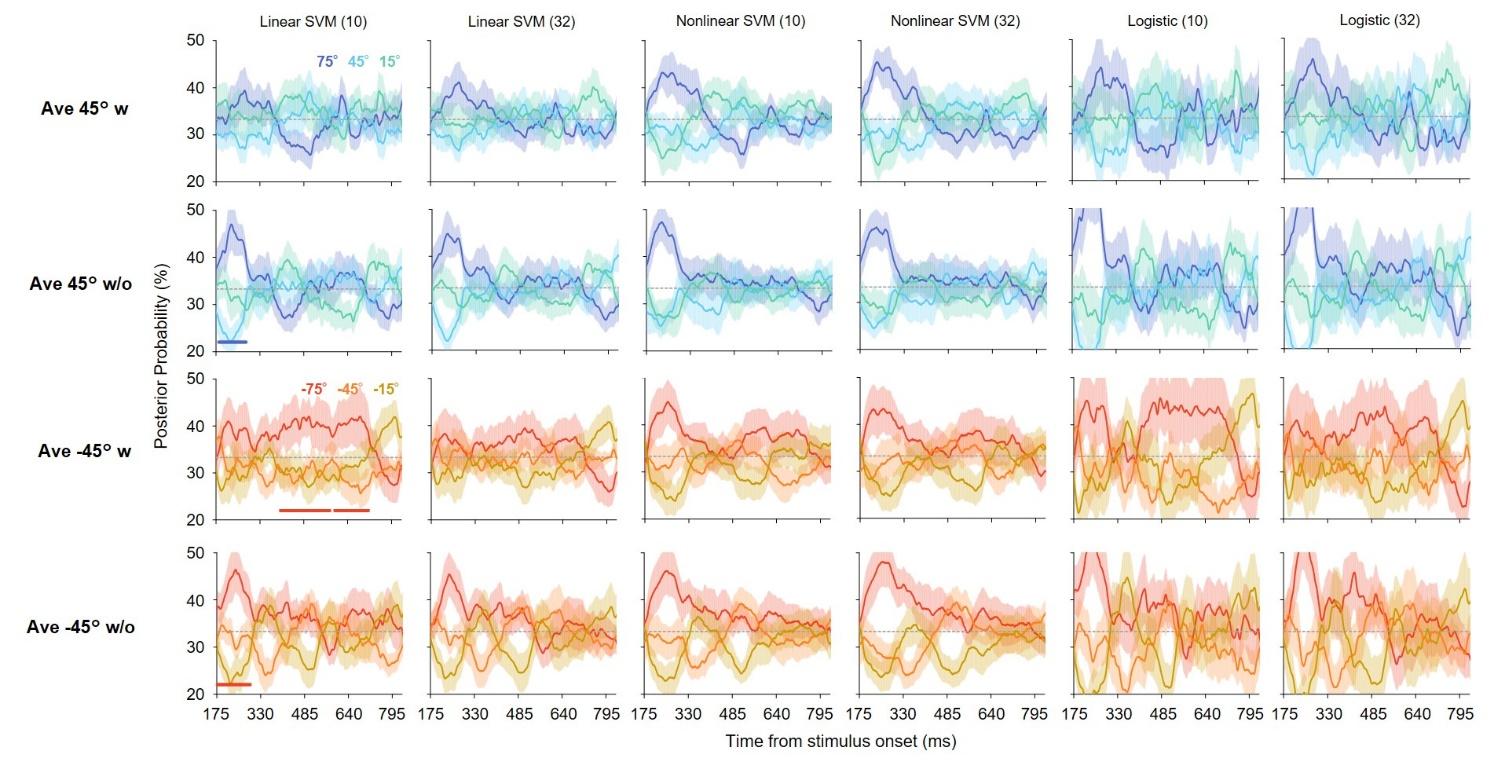


**Supplementary Figure S4.** Time courses of posterior probabilities of orientations included in the stimulus sets in Experiment 2A for different decoder types and number of electrodes used to construct decoders (in parentheses; 10 or 32) The panels in the left column are the same as those in Supplementary Fig. S3. The probability profiles were qualitatively similar across different types of decoders and electrodes. All other conventions are the same as in Supplementary Fig. S3.


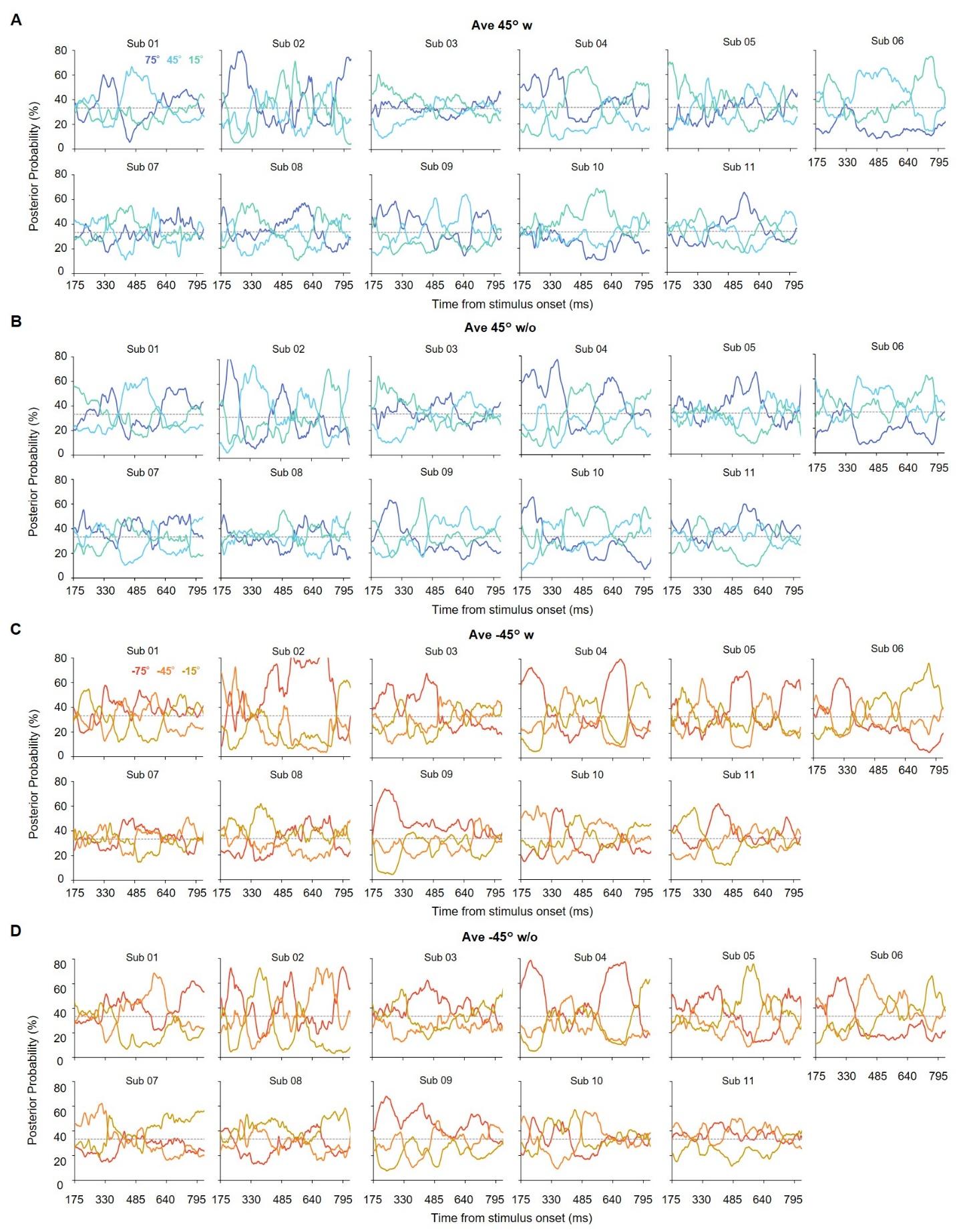


**Supplementary Figure S5.** Time courses of posterior probabilities in Experiment 2A for 11 participants (sub 01-11). Each panel **(A-D)** shows the results for each stimulus set. Different temporal profiles of probabilities (i.e., the relative representational strength of the different orientations) across participants was observed. All other conventions are the same as in Supplementary Fig. S3.

**3 References**

Cant, J. S., & Xu, Y. (2020). One bad apple spoils the whole bushel: The neural basis of outlier processing. *NeuroImage*, *211*, 116629.

Cichy, R. M., Ramirez, F. M., & Pantazis, D. (2015). Can visual information encoded in cortical columns be decoded from magnetoencephalography data in humans? *NeuroImage*, *121*, 193–204.

de Gardelle, V., & Summerfield, C. (2011). Robust averaging during perceptual judgment. *Proceedings of the National Academy of Sciences of the United States of America*, *108*(32), 13341–13346.

Epstein, M. L., Quilty-Dunn, J., Mandelbaum, E., & Emmanouil, T. A. (2020). The outlier paradox: The role of iterative ensemble coding in discounting outliers. *Journal of Experimental Psychology. Human Perception and Performance*, *46*(11), 1267–1279.

Haberman, J., & Whitney, D. (2010). The visual system discounts emotional deviants when extracting average expression. *Attention, Perception & Psychophysics*, *72*(7), 1825–1838.

Lau, J. S.-H., & Brady, T. F. (2018). Ensemble statistics accessed through proxies: Range heuristic and dependence on low-level properties in variability discrimination. *Journal of Vision*, *18*(9), 3.

Li, V., Herce Castañón, S., Solomon, J. A., Vandormael, H., & Summerfield, C. (2017). Robust averaging protects decisions from noise in neural computations. *PLoS Computational Biology*, *13*(8), e1005723.

Pantazis, D., Fang, M., Qin, S., Mohsenzadeh, Y., Li, Q., & Cichy, R. M. (2018). Decoding the orientation of contrast edges from MEG evoked and induced responses. *NeuroImage*, *180*(Pt A), 267–279.

Pascucci, D., Ruethemann, N., & Plomp, G. (2021). The anisotropic field of ensemble coding. *Scientific Reports*, *11*(1), 8212.

Rich, E. L., & Wallis, J. D. (2016). Decoding subjective decisions from orbitofrontal cortex. *Nature Neuroscience*, *19*(7), 973–980.

Tiurina, N. A., Markov, Y. A., Whitney, D., & Pascucci, D. (2024). The functional role of spatial anisotropies in ensemble perception. *BMC Biology*, *22*(1), 28.
